# Vaginal Neoplasm in a Breast Cancer Survivor

**DOI:** 10.31729/jnma.9038

**Published:** 2025-06-30

**Authors:** Baishali Roy, Ruchi Rathore, Seema Singhal, Sholanki Halder, Nilanchi Singh

**Affiliations:** 1 Department of Obstetrics and Gynaecology, All India Institute of Medical Sciences, New Delhi, India; 2 Department of Pathology, All India Institute of Medical Sciences, New Delhi, India

**Keywords:** *carcinoma breast*, *invasive ductal carcinoma*, *vaginal metastasis*

## Abstract

According to GLOBOCAN 2022 data, Carcinoma breast is the second most common malignancy worldwide after lung and ranks 4^th^ in mortality worldwide. Breast cancer can metastasize to various organs. The incidence of vaginal metastasis in carcinoma breast is 1-2%. Here we present a patient with post menopausal bleeding with vaginal mass. She had a history of Triple negative carcinoma left breast 3 years back, treated by Modified Radical Mastectomy followed by chemo radiotherapy. Vaginal biopsy was suggestive of a metastatic carcinoma breast. MRI and PET CT showed isolated vaginal growth. Due to poor performance status and multiple medical comorbidities, decision was taken to treat her with single agent Paclitaxel 3weekly until disease progression. This case report points out the necessity for thorough gynaecological examination in a cancer survivor, either via clinical examination, routine PAP smear or imaging.

## INTRODUCTION

According to GLOBOCAN 2022 data, carcinoma breast is the second most common malignancy worldwide after lung and ranks fourth in mortality worldwide.^1^ Metastases to the genital organs are less common, however metastasis to ovaries are common as compared to vagina.^2^. A review by Mazur et al.^3^ reported that among 149 cases of extragenital tumors metastasizing to the female genital tract, only 3 cases (approximately 2%) involved the vagina originating from breast cancer. Here, we report a case of breast cancer survivor who presented with a vaginal growth.

## CASE REPORT

**Figure 1:**
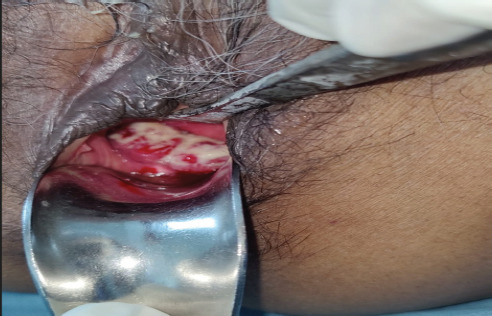
Vaginal mass on speculum examination

Our patient is a 60-year-old, postmenopausal lady with complaints of post-menopausal bleeding for six months. Bleeding was irregular, not associated with foul smelling discharge or pain abdomen. She is postmenopausal for 17 years. She had a history of triple negative (ER, PR, HER 2 neu negative) invasive ductal carcinoma of left breast, stage pT3bN0M0, for which she underwent left modified radical mastectomy. She received four cycles adjuvant therapy of injection adriamycin and cyclophosphamide, followed by four cycles of three-weekly paclitaxel in the year 2021. This was followed by radiation to the chest wall, though we don't have her treatment details. She was disease free for three years. She is a known case of type 2 diabetes mellitus, dilated cardiomyopathy and interstitial lung disease, since 14 years. Examination revealed a 3 × 3 cm growth on the anterior vaginal wall, with irregular margins fixed to the adjoining tissues (Figure 1).

**Figure 2:**
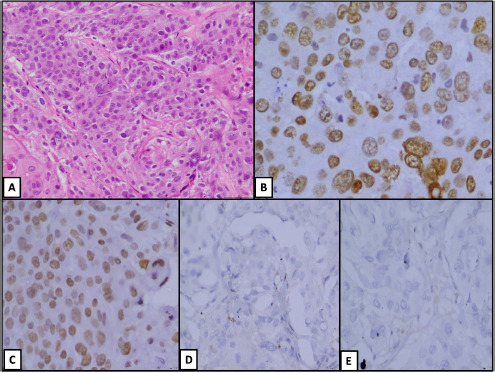
A: Microscopy image shows nests of tumor cells infiltrating the vaginal stroma (HE;200X). B: Tumor cells are immunopositive for GATA 3 and TRPS (C) while are immmunonegative for ER (D) and Her2/nue (E) on immunohistochemistry (IHC;400X)

Incisional biopsy from the mass revealed features suggestive of metastatic invasive adenocarcinoma, tumor cells positive for GATA 3, TPRS1, negative for ER, PR and HER 2 neu (Figure 2).

MRI of whole abdomen and pelvis was suggestive of polypoidal vaginal mass lesion of size 1.8x4.2x3.4 cm, in upper and mid vagina with posterior paravaginal extension. Pelvic lymphadenopathy (9X9 mm in right internal iliac and 8x8 mm in right obturator) were noted. PET-CT Whole Body showed no FDG avid lesion in the breast, chest wall, ribs or elsewhere. Soft tissue mass lesion noted involving upper half of vagina (4x2.4 cm), superiorly involving uterine cervix, anteriorly abutting the posterior wall of urinary bladder with preserved fat planes, though there were no bladder symptoms. With the working diagnosis of a recurrent carcinoma breast with vaginal metastasis, she was planned for further management. Due to poor performance status and multiple medical comorbidities, a multi-disciplinary meeting was conducted and decision was taken to treat her with palliative intent with three weekly single agent paclitaxel, until disease progression. Patient's age and poor performance status were the reasons for choosing single agent therapy over the multiagent ones.

## DISCUSSION

Few cases have been reported about carcinoma breast metastasizing to genital tract. The common sites of distant metastases from breast cancer are lung, bone, liver, and supraclavicular lymph nodes.^2^ Vagina is a rare site of recurrence and metastasis. Metastatic vaginal adenocarcinomas are most commonly found in the uterus and rarely in the breast.

Yan et al. reported a 50-year-old lady with triple-negative carcinoma of the left breast who underwent modified radical mastectomy and adjuvant chemotherapy. A year later, she developed chest wall and sternal metastasis, was treated with further chemotherapy and radiotherapy, and later presented with a vaginal mass. Histopathology confirmed breast origin. She developed widespread metastases within nine months and expired.^4^ In contrast, our patient had no local recurrence in the breast or chest wall before developing the vaginal lesion.

Khadija et al. described a 70-year-old with cT4d N0Mx lobular carcinoma, ER and PR 100%, HER2 negative, and loss of E-Cadherin. Imaging showed a bulky uterus with thickened endometrium. Vaginal biopsy showed suspicious cells with CK7 positivity, partial ER and PR positivity, and HER2 negative, consistent with metastasis.^5^ Our case differed in histology and hormonal status.

Fillopo et al. presented a 54-year-old with previous lobular carcinoma of breast, who later presented with vaginal bleeding. PET-CT revealed isolated vaginal recurrence. Vaginectomy confirmed breast metastasis. She later underwent hysterectomy and bilateral salpingo-oophorectomy, which revealed micrometastasis in the uterus and ovaries.^6^ As in our case, recurrence was isolated to the vagina, but unlike her, our patient was a poor surgical candidate due to comorbidities. Gynecological evaluation is also important in ruling out vulvovaginal atrophy, a frequent issue in breast cancer survivors on aromatase inhibitors.

Aarathi R et al. described a lobular carcinoma metastasizing to the cervix, highlighting the need for cervical screening in breast cancer follow-up.^7^

Treatment of vaginal metastatic lesions ranges from surgery to chemotherapy or radiotherapy. Surgery is an option in resectable disease, but given that recurrent carcinoma breast is systemic, chemotherapy is crucial. Poor prognosis and lack of symptoms suggest the importance of routine gynecological exams in survivors, especially if symptomatic. An important prognostic factor for patients with vaginal metastases from breast cancer is the presence of secondary lesions in other organs.^8^

Our patient, due to age, comorbidities, and advanced local disease, was best managed with systemic chemotherapy. Vaginal metastasis reflects hematogenous spread. Differentiating it from primary vaginal carcinoma is challenging. Primary vaginal cancers, usually squamous cell carcinoma (80-90%), affect the upper posterior wall, while secondary tumors mirror the histology of their primary origin.^9^ IHC was vital in diagnosis in our case.

## CONCLUSIONS

Reported cases of vaginal metastases in carcinoma breast are sparse, more so, in recurrent setting. Carcinoma breast metastasizing to vagina is often mistaken as a primary vaginal carcinoma. For isolated vaginal metastasis, approach is surgical removal with or without chemo radiotherapy.

Metastasis of carcinoma breast to genital tract, emphasizes on the importance of routine gynaecological screening in cancer patients and the fact that patients should keep a low threshold in seeking medical help if any vaginal symptoms appear.
